# Reply to: Epistaxis as a complication of high-flow nasal cannula therapy in adults

**DOI:** 10.5935/0103-507X.20220047resp-en

**Published:** 2022

**Authors:** Viviane Cordeiro Veiga, Lígia Maria Coscrato Junqueira Silva, Érica Regina Ribeiro Sady, Israel Silva Maia, Alexandre Biasi Cavalcanti

**Affiliations:** 1 Intensive Care Unit, BP-A Beneficência Portuguesa de São Paulo - São Paulo (SP), Brazil.; 2 Research Institute, HCor-Hospital do Coração - São Paulo (SP), Brazil.

## TO THE EDITOR

We appreciate the comments by Drs Nair and Esquinas to our article,^(1)^ to which we provide clarifications.

First, according to the institutional protocol, the use of high-flow nasal cannula (HFNC) therapy was contraindicated in patients with predominant mouth breathing, considering its ineffectiveness.^(2)^

Second, all seven patients with epistaxis reported in our case series were evaluated and managed by the institution´s otorhinolaryngology team, including an exploration of the nasal cavity utilizing anterior rhinoscopy. Epistaxis was unilateral in five patients and bilateral in two patients. The Epistaxis Severity Score could not be calculated because it is not applicable to isolated acute epistaxis. Among the 7 patients with epistaxis associated with HFNC use, one case was more severe and needed to be treated with an epistaxis-specific device (Rapid Rhyno®). Two other cases were controlled with topical epinephrine. No patients needed chemical or electrical cauterization.

Third, the Vapotherm® technical manual indicates that the temperature of the delivered gas mixture is accurate within plus or minus 2ºC of the programmed temperature.^(3)^ However, the manufacturer states that the temperature to which patients are exposed depends not only on the temperature titration in the device but also on the titrated flow rate and the ambient temperature. In our institution, the ambient temperature is controlled between 20ºC and 22ºC. We did not find a study that evaluated the accuracy of temperature and humidification in different scenarios of flow or ambient temperatures for the HNFC device used in our patients. Additionally, as the use of HFNCs in the patients whose cases were reported was part of regular clinical practice, there were no measurements of the accuracy of the temperature and humidification of the gas mixture delivered to patients.
